# NR2F1 and mTORC1 provide the bridge between melanoma dormancy and therapeutic resistance

**DOI:** 10.1172/JCI197764

**Published:** 2025-09-16

**Authors:** Narsimha Mamidi, Swadesh K. Das, Paul B. Fisher

**Affiliations:** 1 Department of Cellular, Molecular and Genetic Medicine,; 2 VCU Institute of Molecular Medicine, and; 3 VCU Massey Comprehensive Cancer Center, Virginia Commonwealth University, School of Medicine, Richmond, Virginia, USA.

## Abstract

Cutaneous melanoma (CM) is known for its aggressive behavior, high metastatic potential, and poor prognosis. Mutations in the *BRAF* gene are common in CM, and patients with BRAF-mutant melanoma often respond well to combined inhibition of BRAF and MEK (BRAFi + MEKi). Although BRAFi + MEKi therapy provides clinical efficacy, the response durability is limited by persistent drug-tolerant residual cells, culminating in relapse. In this issue of the *JCI*, Tiago et al. confirmed that NR2F1, a dormancy-associated transcription factor, is a key determinant of therapeutic resistance in melanoma. NR2F1 expression was elevated in transcriptomic datasets from patients with minimal residual disease, and in murine and human melanoma models, NR2F1 overexpression reduced therapeutic efficacy and suppressed tumor proliferation and invasion while sustaining mechanistic target of rapamycin complex 1 (mTORC1) transcriptional regulation of relevant genes. Combining BRAFi + MEKi with the mTORC1 inhibitor rapamycin effectively targeted these resistant melanoma cells, suggesting a potential path forward for targeting NR2F1 and mTORC1 signaling in patients with CM.

## Challenges in cutaneous melanoma treatment

Melanoma represents the most aggressive and lethal form of skin cancer. Its global incidence is rising, from 325,000 new cases and 57,000 deaths reported in 2020 to 510,000 cases and 96,000 deaths projected annually by 2040, and reflects marked geographical variation (1, 2). Early detection and intervention are critical to melanoma outcomes, as prognosis strongly correlates with tumor depth, which progresses over time. Current melanoma treatments, including immune checkpoint inhibitors and MAPK-targeted therapies, face obstacles and limitations due to innate and acquired resistance (3–5). Therapy combining BRAF-V600E inhibitors with MAPK kinase inhibitors (BRAFi + MEKi) promotes tumor shrinkage in patients with BRAF mutation (3). Still, this intervention often fails within a 14-month treatment period due to resistance (6). Moreover, BRAFi + MEKi resistance intensifies in an aged tumor microenvironment (TME) (7), and this targeted approach has reduced efficacy in older mice (8).

**Figure 1:**
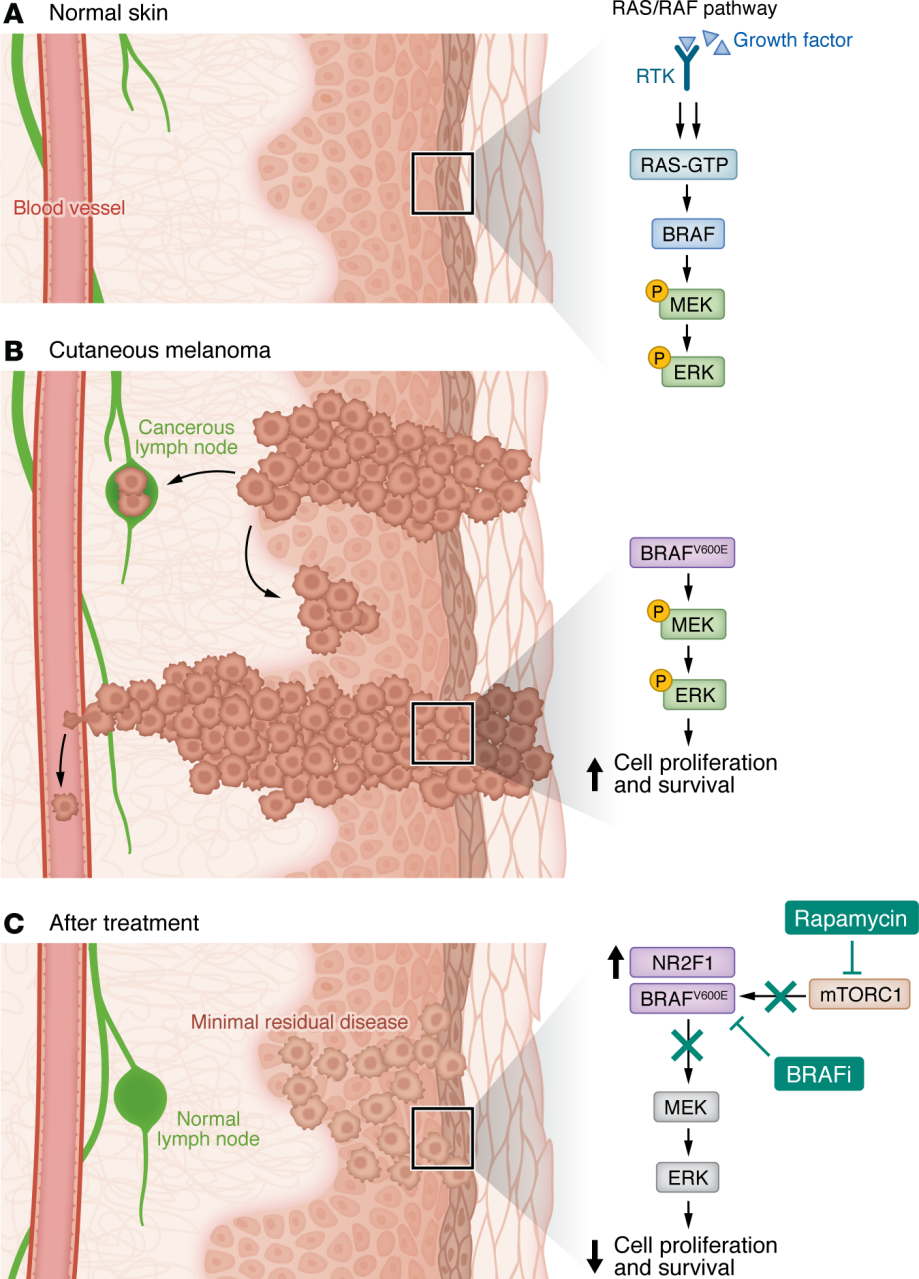
Progression from normal skin to cutaneous melanoma and the molecular mechanisms underlying the effects of targeted therapy demonstrated by Tiago et al. (**A**) Normal skin: A cross-section shows epidermis, dermis, and underlying tissues. In healthy melanocytes, the RAS/RAF pathway is initiated by growth factor binding to its receptor, RTK, which activates RAS-GTP, which in turn activates BRAF. Activated BRAF phosphorylates MEK, which then phosphorylates ERK, leading to normal cell proliferation and survival. (**B**) Cutaneous melanoma: The cross-section shows development of melanoma, with abnormal melanocyte proliferation invading deeper layers and metastatic spread to lymph nodes. In melanoma cells, mutant, constitutively active BRAF (commonly the BRAF-V600E mutation depicted here) leads to persistent phosphorylation and activation of MEK and ERK, resulting in uncontrolled cell proliferation and survival (oncogenesis). (**C**) After treatment: In post-treatment skin, despite reduced tumor presence and normal lymph nodes, slow-cycling, drug-resistant cells can persist (collectively referred to as MRD). Tiago and colleagues identified upregulation of NR2F1 in these cells (19). Targeted inhibition of mutant BRAF using BRAFi disrupts the pathway, halting MEK and ERK phosphorylation, thus reducing cell proliferation and survival. Tiago et al.’s findings revealed that additional inhibition of mTORC1 by rapamycin (an mTOR inhibitor), which binds selectively to mTORC1, further impaired cell growth and proliferation.

BRAF, a serine/threonine kinase, is the primary RAF-family activator of MAPK/ERK signaling. Within the RAF family, BRAF is distinguished by its high basal activity and potent RAS-responsive attributes, which likely drive its frequent oncogenic mutations (9). In melanoma, approximately 50% of patients harbor BRAF alterations, with more than 90% of these alterations targeting codon 600 (predominantly V600E, a T1799A valine-to-glutamate substitution) (9). In normal skin cells, growth factors activate receptor tyrosine kinases (RTKs), triggering RAS-GTP–dependent BRAF activation and MEK/ERK signaling, thereby promoting controlled proliferation (Figure 1A). In cutaneous melanoma, mutant BRAF constitutively activates this pathway, driving uncontrolled growth and lymph node metastasis (Figure 1B).

However, treatment strategies that have enduring efficacy in patients with melanoma remain an unmet clinical need. Many patients who initially respond well to BRAFi + MEKi treatment ultimately relapse, an outcome driven by a small population of residual cancer cells collectively referred to as minimal residual disease (MRD). MRD consists of slow-cycling, drug-tolerant persister (DTP) cells (10, 11), some of which are invasive and participate in recurrence (12). DTP cells exhibit traits of dormancy (13) driven by mechanisms corresponding to AXL overexpression, MITF/SOX10 loss, and stress pathways including p38 and PERK (14–16). Across cancers, disseminated DTP cells show similar behaviors (17), and nuclear receptor subfamily 2 group F member 1 (NR2F1) has been reported as a dormancy regulator in other cancers (18).

The transient nature of therapeutic responses and frequency of relapse highlight the prerogative to find targets to confront MRD. In this issue of the *JCI*, Tiago et al. identified NR2F1 as being highly expressed in invasive DTPs and MRD xenografts following BRAFi + MEKi treatment, shedding light on the role of NR2F1 in mediating drug tolerance and persistence of invasive residual disease in *BRAF*-mutant melanoma (19).

## NR2F1 overexpression links melanoma dormancy to relapse

Tiago and colleagues’ analysis of published single-cell and bulk transcriptomics datasets confirmed that NR2F1 was highly expressed in BRAFi + MEKi–treated patients with melanoma and BRAF-mutant melanoma patient–derived xenografts, particularly in invasive MRD, as well as in preclinical cell models. Overexpression of NR2F1 in the BRAF-V600E 1205Lu, WM793, and A375 melanoma cell lines reduced the tumor growth–inhibiting effects of BRAFi + MEKi by enhancing proliferation, survival, and invasion in vitro while accelerating tumor relapse in vivo, partly through sustained mechanistic target of rapamycin complex 1 (mTORC1) signaling. These findings revealed that NR2F1 promotes a dormant yet invasive phenotype, enabling tumor cells to evade targeted therapy and cultivating acquired resistance (19). These genotypic and phenotypic changes provide a mechanism by which a subset of therapy-treated melanoma (and potentially other tumor cell types) can avoid elimination by targeted therapies and fuel tumor latency and MRD (19).

Notably, Tiago and colleagues also reported that melanomas in aged mice exhibited elevated NR2F1 levels (19), aligning with prior observations that aged mice showed a poorer response to BRAFi + MEKi than did their younger counterparts (8, 19). A similar elevation of NR2F1 levels in melanoma cells conditioned with skin fibroblasts from older, healthy donors suggested that the aged TME contributed to NR2F1 expression. NR2F1 knockdown in aged melanoma models improved drug efficacy and delayed disease progression. Combining BRAFi + MEKi with mTOR inhibitors (e.g., rapamycin) suppressed NR2F1-driven MRD and delayed relapse, suggesting that targeting NR2F1 or downstream pathways in DTP cells is a potentially viable strategy to overcome dormancy-like resistance to melanoma therapy. Therapeutic inhibition of mTORC1 by rapamycin and blockade of BRAF/MEK signaling constitute a targeted intervention to suppress tumor progression (Figure 1C). The key to the intervention’s efficacy will be to either directly or indirectly destroy these latent DTP tumor cells in MRD or prevent them from crossing the bridge from latency to relapse into an active disease state with pathogenic consequences.

## Clinical implications

The findings of Tiago et al. imply that, clinically, NR2F1 marks invasive, drug-tolerant melanoma cells that persist after therapy, driving relapse, and that cotargeting mTORC1 and MAPK pathways may improve outcomes, especially in older patients with resistant disease. This work also links aged microenvironments to NR2F1 upregulation, providing a plausible explanation for poorer responses in aging populations and uncovering potentially new therapeutic opportunities.

NR2F1 expression could serve as a biomarker to identify patients with melanoma at higher risk of relapse after BRAFi + MEKi therapy, particularly those with invasive or drug-tolerant disease. Additionally, monitoring its levels in circulating tumor cells (CTCs) or biopsies during treatment may help predict therapeutic resistance. Older patients, who often respond poorly to BRAF and MEK inhibitors and have higher NR2F1 levels, may benefit from early intervention with mTOR inhibitors to delay resistance, and clinical trials could test triplet therapy (BRAFi + MEKi + mTOR inhibitor) in this population or in a broader population with high NR2F1 expression. Additionally, NR2F1-driven MRD represents a reservoir for relapse, suggesting that combining BRAFi + MEKi with dormancy-disrupting agents like mTOR inhibitors may improve progression-free survival, with intermittent dosing of mTOR inhibitors explored to reduce toxicity while maintaining long-term disease control. Finally, strategies to modulate fibroblast-secreted factors (e.g., SFRP2, RARRES2) in older patients may help suppress NR2F1 and enhance BRAFi + MEKi efficacy by overcoming the effect of the aged TME.

## Unanswered questions and future directions

Several key unanswered questions and future directions emerge from the study of NR2F1 in melanoma therapy resistance (19). Mechanistically, it remains unclear whether NR2F1 sustains mTORC1 signaling through direct binding to mTOR pathway gene promoters (e.g., *PDK1*, *HSPD1*) or via intermediate regulators, and whether NR2F1 relies on cofactors to exert its prosurvival effects. What drives NR2F1 upregulation in the aged TME is also unknown, but may include contributions from fibroblast-derived factors (e.g., Wnt5A, SFRP2), epigenetic changes, and/or retinoic acid signaling. Additionally, NR2F1’s relationship with other resistance mechanisms such as AXL-, SOX9-, or MITF-low states and its potential cooperation with epigenetic reprogramming to maintain dormancy require further investigation. Considering the potential importance of NR2F1 in regulating tumor latency and melanoma MRD, diving even deeper into mechanism(s) of action will provide additional opportunities to develop creative and potentially effective therapies in treating residual and therapy-refractive disease. The “holy grail” would be the conversion of transient clinical responses into stable clinical outcomes.

Therapeutically, it remains to be determined whether NR2F1 can be directly targeted in melanoma with small-molecule inhibitors or degraders. Notably, NR2F1 agonists have been investigated in other cancer types (e.g., breast and prostate cancer models) to induce tumor cell dormancy, but their effectiveness and translational potential in melanoma models are currently unexplored (19). Future studies are warranted to clarify whether strategies that modulate NR2F1 activity, either through inhibition or agonism, could confer clinical benefit in the context of BRAF-mutant melanoma.

Optimization of mTOR inhibitor regimens is another key area for exploration, including determining whether intermittent dosing schedules (such as with rapamycin) can offer advantages over continuous administration and evaluating whether selective mTORC1 inhibition (e.g., rapamycin) or dual mTORC1/2 inhibitors (e.g., AZD2014) provide superior efficacy or tolerability in melanoma models.

The role of NR2F1 in modulating immunotherapy responses also warrants investigation, including whether NR2F1-high melanomas are more responsive to PD-1 or CTLA-4 blockade, or if NR2F1 influences T cell infiltration (5). Clinically, it is unknown whether NR2F1 plays a role in resistance in other BRAF-mutant cancers (e.g., colorectal, thyroid) or dormancy in solid tumors like breast or prostate cancer. Finally, future studies should assess whether NR2F1 levels could guide treatment decisions, such as initiating triplet therapy earlier in patients with high NR2F1 expression and whether liquid biopsies could detect NR2F1-expressing CTCs to monitor MRD. Additional key factors in restraining tumor progression and in developing and escaping from tumor dormancy may involve interactions (via direct contact or secreted factors) between tumor cells and cells in the TME (20). Potentially, targeting NR2F1 and mTOR in both compartments, i.e., the tumor and TME, may amplify the therapeutic effect of future targeted inhibitors.

## Conclusions

Tiago et al.’s findings bridge the gap between tumor dormancy and drug resistance, emphasizing NR2F1 as a linchpin in melanoma persistence, particularly in older patients. Their work aligns with growing evidence that residual disease is not merely passive but actively adapts to therapy. The study also emphasizes the effect of aging on treatment efficacy, a critical consideration given melanoma’s prevalence in older adults. The insights from this and future work in this area offer the potential to not only further understand the underpinnings of cancer progression and latency, but also the role of therapy in galvanizing a link between therapy resistance, development, and maintenance of latent disease and the process of aging.

In addition to identifying NR2F1 as a key molecular driver of drug tolerance in melanoma, Tiago and colleagues have highlighted mTOR inhibition as a promising strategy to delay relapse and provided strong evidence supporting NR2F1 as both a marker and mediator of residual disease in BRAF-mutant melanoma. Their findings emphasize the importance of targeting the persistent cells responsible for recurrence, moving beyond initial tumor response metrics. As our understanding of tumor heterogeneity and the microenvironment deepens (19), disrupting the NR2F1 axis may offer a path to more durable remissions. However, critical questions remain about NR2F1’s regulatory mechanisms, its role in therapy resistance across cancer types, and how best to target it therapeutically. Future research should focus on three priorities: (a) defining NR2F1’s molecular signaling pathways, (b) advancing clinical trials of mTOR/BRAF/MEK combination therapies, and (c) developing biomarkers to identify patients who are most likely to benefit from NR2F1-targeted approaches. The above strategies have the potential to improve outcomes for patients with melanoma facing residual disease and treatment resistance.

## Version 1 09/16/2025

Electronic publication

## References

[1] Naik PP (2021). Cutaneous malignant melanoma: a review of early diagnosis and management. World J Oncol.

[2] Arnold M (2022). Global burden of cutaneous melanoma in 2020 and projections to 2040. JAMA Dermatol.

[3] Michielin O (2020). Evolving impact of long-term survival results on metastatic melanoma treatment. J Immunother Cancer.

[4] Bhave P (2021). Melanoma recurrence patterns and management after adjuvant targeted therapy: a multicentre analysis. Br J Cancer.

[5] Wang XY (2011). Blockade of cytotoxic T-lymphocyte antigen-4 as a novel therapeutic approach for advanced melanoma. Expert Opin Pharmacother.

[6] Savola P (2020). Clinical implications of acquired BRAF inhibitors resistance in melanoma. Int J Mol Sci.

[7] Fane M, Weeraratna AT (2020). How the ageing microenvironment influences tumour progression. Nat Rev Cancer.

[8] Chhabra Y (2024). Sex-dependent effects in the aged melanoma tumor microenvironment influence invasion and resistance to targeted therapy. Cell.

[9] Wellbrock C (2004). The RAF proteins take centre stage. Nat Rev Mol Cell Biol.

[10] Swayden M (2020). Tolerant/persister cancer cells and the path to resistance to targeted therapy. Cells.

[11] Rambow F (2018). Toward minimal residual disease-directed therapy in melanoma. Cell.

[12] Perego M (2018). A slow-cycling subpopulation of melanoma cells with highly invasive properties. Oncogene.

[13] Risson E (2020). The current paradigm and challenges ahead for the dormancy of disseminated tumor cells. Nat Cancer.

[14] Glasheen MQ (2023). Targeting upregulated cIAP2 in SOX10-deficient drug tolerant melanoma. Mol Cancer Ther.

[15] Capparelli C (2022). Targeting SOX10-deficient cells to reduce the dormant-invasive phenotype state in melanoma. Nat Commun.

[16] Aguirre-Ghiso JA (2003). ERK(MAPK) activity as a determinant of tumor growth and dormancy; regulation by p38(SAPK). Cancer Res.

[17] Mikubo M (2021). Mechanism of drug tolerant persister cancer cells: the landscape and clinical implication for therapy. J Thorac Oncol.

[18] Sosa MS (2015). NR2F1 controls tumour cell dormancy via SOX9- and RARβ-driven quiescence programmes. Nat Commun.

[19] Tiago M (2025). Elevated NR2F1 underlies the persistence of invasive disease after treatment of BRAF-mutant melanoma. J Clin Invest.

[20] Madan E (2022). Cell competition in carcinogenesis. Cancer Res.

